# Endophytic *Paenibacillus polymyxa* LMG27872 inhibits *Meloidogyne incognita* parasitism, promoting tomato growth through a dose-dependent effect

**DOI:** 10.3389/fpls.2022.961085

**Published:** 2022-09-14

**Authors:** Richard Raj Singh, Wim M. L. Wesemael

**Affiliations:** ^1^Department of Plants and Crops, Faculty of Bioscience Engineering, Ghent University, Ghent, Belgium; ^2^Plant Unit, Flanders Research Institute for Agriculture, Fisheries and Food (ILVO), Merelbeke, Belgium

**Keywords:** root-knot nematode, *Solanum lycopersicum*, hatching, biocontrol, pest management

## Abstract

The root-knot nematode, *Meloidogyne incognita*, is a major pest in tomato production. *Paenibacillus polymyxa*, which is primarily found in soil and colonizing roots, is considered a successful biocontrol organism against many pathogens. To evaluate the biocontrol capacity of *P. polymyxa* LMG27872 against *M. incognita* in tomato, experiments were conducted both *in vitro* and *in vivo*. A dose-response effect [30, 50, and 100% (10^8^ CFU/mL)] of bacterial suspensions (BSs) on growth and tomato susceptibility to *M. incognita* with soil drenching as a mode of application was first evaluated. The results show that the biological efficacy of *P. polymyxa* LMG27872 against *M. incognita* parasitism in tomato was dose-dependent. A significantly reduced number of galls, egg-laying females (ELF), and second-stage juveniles (J2) were observed in BS-treated plants, in a dose-dependent manner. The effect of *P. polymyxa* on tomato growth was also dose-dependent. A high dose of BSs had a negative effect on growth; however, this negative effect was not observed when the BS-treated plants were challenged with *M. incognita*, indicating tolerance or a defense priming mechanism. In subsequent *in vivo* experiments, the direct effect of BSs was evaluated on J2 mortality and egg hatching of *M. incognita*. The effect of BS on J2 mortality was observed from 12 to 24 h, whereby *M. incognita* J2 was significantly inhibited by the BS treatment. The effect of *P. polymyxa* on *M. incognita* egg hatching was also dependent on the BS dose. The results show a potential of *P. polymyxa* LMG27872 to protect plants from nematode parasitism and its implementation in integrated nematode management suitable for organic productions.

## Introduction

*Meloidogyne* spp. root-knot nematodes (RKNs) are economically important plant-parasitic nematodes (PPNs) with a wide host range (Moens et al., 2009; Jones et al., 2013). They cause gall formation or root knots, disrupting the host plant physiology, thus resulting in crop losses. In some crops, damage caused by RKNs can be extremely high and reach up to 100% (Wesemael et al., 2011). *Meloidogyne incognita* is one of the most serious belowground pests of tomato, one of the most widely cultivated crops in the world (Seid et al., 2015). In Flanders, RKNs are a major constraint for organic protected fruit vegetable production. While few nematicides are used in the control of this nematode, chemical nematicides are generally non-environmental-friendly and are no option for organic production. Thus, there is a pressing need to promote environment-friendly crop protection products for sustainable agriculture. Microorganisms such as endophytic bacteria that live within a plant at least part of their life cycle may exert beneficial effects on plant growth or health, either directly or indirectly (Vejan et al., 2016). They employ a variety of mechanisms to protect their host plant from biotic and abiotic stress, including the production of antimicrobial metabolites and toxic volatile compounds (VOCs), enhancing tolerance, and activating induced systemic response (ISR) (Gómez-Lama Cabanás et al., 2014; Timmusk et al., 2015; Li and Chen, 2019; Chávez-Ramírez et al., 2020; Mengistu, 2020; Dudeja et al., 2021). They have beneficial effects such as promoting growth *via* biofertilization, such as fixation of atmospheric nitrogen and converting it to ammonia for plant uptake, phosphate solubilization, or sulfur oxidation (Grayston and Germida, 1991; Turan et al., 2016; Walia et al., 2017; Emami et al., 2020; Langendries and Goormachtig, 2021). Some isolates also can supply or trigger production of phytohormones (Glick et al., 2007; Ek-Ramos et al., 2019; Finkel et al., 2020).

The use of endophytic bacteria has shown promising effects on several crops against PPNs, such as control of *M. incognita* in tomato (Munif et al., 2013; Li et al., 2019), sponge gourd (Abd El-Aal et al., 2021), cucumber (Hallmann et al., 1998), potato (Hallmann et al., 2001), and coffee (Hoang et al., 2020); *M. javanica* in tomato (Siddiqui and Ehteshamul-Haque, 2000; Ali Siddiqui and Ehteshamul-Haque, 2001; Siddiqui and Shaukat, 2002a,b) and cotton (Hallmann et al., 1997, 1998, 1999); and control of cyst nematode *Globodera pallida* in potato (Hasky-Guenther et al., 1998; Reitz et al., 2000). Endophytic bacteria colonize root tissues inter- and/or intracellularly and have a mutualistic interaction with host plants (Schulz and Boyle, 2006; Mandyam and Jumpponen, 2015). They colonize the same niche (root tissues) required by RKNs for survival, leading to competition for space and nutrients in the root, which ultimately results in negative effects on RKNs (Backman and Sikora, 2008; Sikora et al., 2008). Therefore, the association of endophytic bacteria throughout the nematode life cycle makes these bacteria excellent candidates for biocontrol strategies (Siddiqui and Shaukat, 2002a). Endophytic bacteria can affect different stages of the nematode life cycle, such as influencing egg hatching, attraction, and recognition behavior in the rhizosphere, possibly by altering the root exudate patterns (Sikora et al., 2008). They are known to control RKNs at pre-plant and during the vegetative plant growth, by colonizing nematode eggshells or the larval cuticle, or through direct penetration (Hallmann et al., 2009).

In Europe, there are few biological nematicides that are currently in use for the control of PPNs, such as (1) BioAct^®^ or MeloCon^®^ with active ingredient fungus *Purpureocillium lilacinum* (Kiewnick and Sikora, 2006; Sikora et al., 2008), and (2) Serenade^®^ and VOTiVO^®^ with active ingredient bacteria *Bacillus amyloliquefaciens* QST713 and *B. firmus* I-1582 (Terefe et al., 2009; Castillo et al., 2013). However, *P. lilacinum* effectiveness against *Meloidogyne* spp. populations was not able to constantly suppress nematode densities under greenhouse conditions (Giné and Sorribas, 2017), and more than one mode of application was necessary for optimum efficacy against *M. incognita* in tomato (Kiewnick and Sikora, 2004).

This study focused on a Gram-positive, rod-shaped bacterium identified as *Paenibacillus polymyxa*, isolate LMG27872 (formerly known as *Bacillus polymyxa*), which resides mainly in the soil, rhizosphere, and plant tissue (Lal and Tabacchioni, 2009). *Paenibacillus polymyxa* is a rod-shaped endospore-forming bacterium and is thermophilic, facultatively anaerobic, neutrophilic, and sporulating with peritrichous flagella for motility and crawling (He et al., 2007; Langendries and Goormachtig, 2021). *Paenibacillus polymyxa* has been described as a successful biocontrol organism against various plant pathogens, including bacteria, fungi, oomycetes, and PPNs (Khan et al., 2008; Son et al., 2009; Cheng et al., 2017; Chávez-Ramírez et al., 2020, 2021). It is known to produce antibiotic compounds and secondary metabolites, such as non-ribosomally synthesized lipopeptides, polymyxins, fusaricidin, sideromycins, macrolactin D, and other VOCs, with direct effects on pathogens or inducing resistance through elicitation of plant systemic defense (Raza et al., 2009; Zhao et al., 2011; Lee et al., 2012; Raza et al., 2015; Lal et al., 2016; Cheng et al., 2017; Krämer et al., 2020; Langendries and Goormachtig, 2021; Mülner et al., 2021). *Paenibacillus polymyxa* is known to secrete siderophores, a known secondary metabolite that scavenges iron, protecting the plants from oxidative stress (Raza et al., 2011; Zhou et al., 2016; Kramer et al., 2020). In addition, it enhances growth *via* production of several plant growth hormones, supplying plants with nitrate/ammonia, and forming biofilms in roots (Timmusk and Wagner, 1999; Timmusk et al., 2005, 2015; Oleńska et al., 2020; Langendries and Goormachtig, 2021). The aim of this study was to evaluate the biocontrol capacity of *P. polymyxa* LMG27872 against the RKN, *M. incognita*, in tomato. Experiments were conducted both *in vitro* and *in vivo*. This research first evaluated a dose-response effect of *P. polymyxa* on tomato susceptibility to nematodes and growth, with soil drenching as a mode of application. The effect of *P. polymyxa* was observed on different stages of nematodes. Furthermore, *in vitro* tests were performed to evaluate the direct effect of *P. polymyxa* on *M. incognita* J2 mortality and hatching.

## Materials and methods

### Plant material and growth conditions

Tomato, *Solanum lycopersicum* cv. Marmande, was used in this study. Seeds were germinated on a sterile potting mixture in a glasshouse (20–26^°^C, 16-h light). At 2 weeks after germination, seedlings were transferred into small pots (200 mL, one seed per pot) containing a heat-sterilized (100^°^C, 8 h) soil/sand mixture (1:1, v/v). Plants were kept in a glasshouse (20–26^°^C, 16-h light) and watered upon requirement.

### Bacterial culture

The endophytic *P. polymyxa* LMG27872 isolate was collected from the rhizosphere of tomato plants in the greenhouse of Flanders Research Institute for Agriculture, Fisheries and Food (ILVO), Merelbeke, Belgium. The isolate was streaked from a Microbank vial (Pro-Lab diagnostics) maintained at –70^°^C onto *Pseudomonas* agar F (PAF) supplemented with 10 g L^–1^ sucrose, instead of glycerol (PAF-S, Becton Dickinson), and incubated for 48 h. The bacterium was grown on agar plates and liquid medium, as illustrated in Hoang et al. (2020). To obtain inoculum, a single colony was transferred into brain heart infusion (BHI; OXOID Ltd., Basingstoke, Hampshire, United Kingdom) liquid medium as pre-culture and incubated overnight in an incubator shaker at 28^°^C continuously shaken at 200 rpm. From this, 1.5 mL of pre-culture bacterium was poured into 500 mL liquid medium and incubated overnight at 28^°^C and 200 rpm. The suspension of bacteria from the main culture was transferred to sterilized centrifuge bottles and centrifuged at 4^°^C for 15 min at 4,000 rpm to form pellets. The supernatant was discarded, and sodium phosphate buffer was added to the pellets; it was gently mixed, and this was considered as a stock solution. The density of the stock solution was determined by using an optical density (OD) spectrophotometer (Thermo Fisher Scientific, United Kingdom) at an absorbance of 600 nm, which was confirmed by plating on PAF-S and calculating the colony-forming unit (CFU/mL) (Beal et al., 2020). An OD of 1 represented a concentration of approximately 1 × 10^8^ CFU mL^–1^, constituting our stock bacterial suspension (BS) (Su et al., 2017). For further use in our experiments, the BS was diluted in PBS at the following concentrations: BS0, BS30, BS50, and BS100%. Fresh BSs were prepared for each experiment.

### Chemical preparation

For nematode parasitism assay (pot experiment), β-aminobutyric acid (BABA) was used as a positive control. The RS BABA racemic mixture was supplied by Eastman, Belgium. A measure of 5 mM BABA was dissolved in water and applied as a soil drench. With this dose, anti-nematode effects have previously been observed on tomato (Oka et al., 1999; Oka and Cohen, 2001; Sahebani and Hadavi, 2009; Cohen et al., 2016) with soil drench as a mode of application. For the *in vitro* assay on J2 mortality (nematicide test) Velum^®^ Prime (Bayer CropScience), a chemical nematicide with active ingredient fluopyram 1.0 μg/mL, was used as positive control.

### Effect of *Paenibacillus polymyxa* LMG27872 on tomato susceptibility to *Meloidogyne incognita*

**FIGURE 1:**
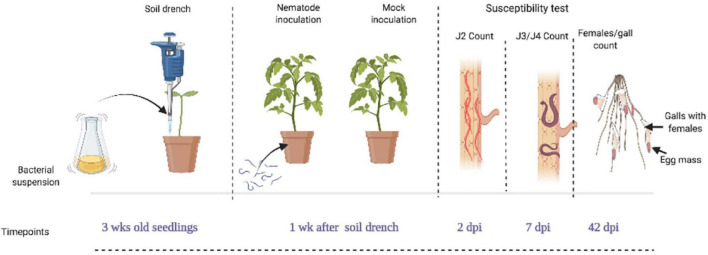
Diagram showing the nematode parasitism experimental setup and timeline for susceptibility test on seedlings. Tomato seedlings of 3 weeks old were soil-drenched with bacterial suspension (BS) at doses of 30, 50, and 100%, while control plants were mock- and buffer-treated (Ctrl and BF) with the positive control, β-aminobutyric acid (BABA). Four weeks after germination (1 week after application of the bacteria), plants were inoculated with 300 *M. incognita* J2 per plant and were analyzed for the total number of galls and egg laying females (ELF) at 42 days post-inoculation (dpi), the number of J2 and J3/J4 stages at 2 and 7 dpi. This figure was created with www.BioRender.com.

For this experiment, 3-week-old tomato seedlings were soil-drenched with bacterial suspension (BS-treated), water (mock-treated), buffer (negative control), and BABA (positive control). The soil was moistened till just below the field capacity and 3 mL of (30, 50 and 100% BS) was applied. After application, the plants were not watered for 24 h to avoid drainage of the BSs. At 4 weeks after germination (1 week after application of BS), each pot was inoculated with 300 freshly hatched J2 of *M. incognita*. Each treatment was replicated eight times for each observation. To evaluate the number of penetrated J2, developed J3/J4, and galls and egg-laying females (ELF) in the nematode-parasitized root, samples were collected at 2, 7, and 42 days post-inoculation (dpi). Figure 1 shows the nematode parasitism experiment setup and timeline for evaluation of tomato susceptibility. To visualize the nematodes inside the root system, staining as described by Byrd et al. (1983) was followed with slight modifications. First, the soil was gently washed off the roots and subsequently washed in bleach (1% NaOCl solution), followed by rinsing. The nematode-parasitized roots were then boiled for 1 min in 0.013% acid fuchsin and 0.8% acetic acid solution. The roots were washed under running tap water and incubated in acidified glycerol. This treatment stains nematodes with a purple color, while the rest of the root system is gradually destained in the acidified glycerol. Plant susceptibility was determined by counting the number of galls and ELF, J2, and J3/J4 stages with the aid of a binocular microscope. The number of ELF was used as criterion to measure nematode reproduction. The nematode parasitism experiment was repeated two times.

### Effect of *Paenibacillus polymyxa* LMG27872 on tomato growth

To evaluate if *P. polymyxa* LMG27872 has an effect on plant phenotype, BS-treated plants inoculated with nematodes (parasitized group), from the previous experiment, were measured for their shoot height (SH), root length (RL), shoot weight (SW), and root weight (RW) at 2 dpi corresponding to 9 days posttreatment (dpt), 7 dpi/14 dpt, and 42 dai/49 dpt, respectively. For each dose and time of evaluation, a set of eight additional plants was grown without nematode inoculation but BS-treated; their growth was evaluated at 9, 14, and 49 dpt. SH and RL were measured using a ruler and Fiji software, an image processing package based on ImageJ (Schindelin et al., 2012). Fresh weight was measured, whereby root and shoot tissues were separated. Tissues were blotted dry and weighed using a digital balance (A&D Weighing BM-200 Ion Analytical Balance). The phenotypic effect of applying *P. polymyxa* LMG27872 on tomato seedlings was also evaluated with an *in vitro* assay according to Khaliluev et al. (2022) with slight modifications. Seeds were surface-sterilized in NaCl and rinsed in water. A total of 10 seeds were germinated in a moist tissue paper in a sterile Petri dish, and 3 days later, 1 mL of a different dose of BS was applied, by immersing the seedling roots in BS for 30 min. The seedlings were then transferred to MS agar media and kept in an incubator at 24^°^C. Control groups were mock-treated or with buffer. At 5 days posttreatment, the seedlings were evaluated for their SH, RL, SW, and RW.

### Effect of *Paenibacillus polymyxa* LMG27872 on hatching of *Meloidogyne incognita* (*in vitro* test)

The effect of different doses of BS (30, 50, and 100%) on hatching of *M. incognita* eggs was tested, as described in Wesemael et al. (2006), with slight modifications. In the control treatments, the BS was replaced by distilled water (mock-treated), buffer was the negative control, and Velum^®^ Prime was the positive control. Egg masses were excised from 6-week-old parasitized tomato plants. Eggs were extracted by cutting heavily galled roots into 1-cm pieces and then vigorously shaken in a 1% NaOCl water solution for 3 min. Single eggs were freed from the egg mass, and approximately 100 individual eggs per replicate were exposed to the different BS doses in embryo dishes. The eggs were kept in the dark in an incubator at 25^°^C. Hatching was monitored with the aid of a binocular microscope after 1, 3, 7, and 14 days. Each treatment was replicated eight times. The number of hatched J2 was counted for each time point, and the relative suppression rate of *M. incognita* egg hatch was calculated over the time course of 14 days, according to Yang et al. (2016). The relative suppression rate was calculated as follows: Relative suppression rate (%) = (No. of J2 in control – No. J2 in treatments)/No. J2 in control × 100.

### Effect of *Paenibacillus polymyxa* LMG27872 on J2 mortality

The direct effect of *P. polymyxa* LMG27872 on J2 mortality was evaluated using 100 freshly hatched J2 that were added into cavity blocks with 1-mL high dose (100%) of BS, with a mock-treated and a positive control (Velum^®^ Prime 1.0 μg/mL). The cavity blocks were placed in the dark at 23^°^C. The J2 motility was monitored by viewing J2 under a dissecting microscope at 6, 12, and 24 h post-incubation. During observation, the immobile J2 were probed with a picking needle; if they were not moving, they were considered dead. After observation, the nematodes were transferred into distilled water to check for recovery. Dead J2 were observed, and mortality was calculated as% of total at respective time point of exposure.

### Evaluation of *Paenibacillus polymyxa* colonization in tomato roots

To evaluate *P. polymyxa* LMG27872 colonization of the treated plants, an assay was performed, as described in Zhao et al. (2015) and Rat et al. (2021), with slight modifications. Then, 3-week-old tomato seedlings were soil-drenched with high-dose 100% BS (since this is where we observed the most efficient control of nematodes), while control plants were mock-treated. Plant roots were investigated for colonization at three different time points after BS treatment, 1, 3, and 7 days. Segments of roots from five plants per treatment were pooled together and were first cleaned with water and then sterile water to remove remaining soil particles and attached epiphytic bacteria. A paint brush was used to gently remove soil particles without creating any wound. The roots were cut into 1–2-cm sections. These sections were further surface-sterilized by sequential immersion in 70% ethanol for 5 min and then rinsed again for 3 min, followed by further sterilization with a solution of 1.4% of NaOCl for 30 min. The root samples were given a final rinse in sterile water for 10 min and then immersed in 2% Na_2_SO_3_ for 10 min to neutralize the effect of bleach. The sterilized roots were placed into a sterile metal mortar, ground to slurry with 0.85% sterile saline, and shaken with a vortex for 1 min. The supernatant was collected for OD measurement. Subsequently, 50 μL of each processed sample suspension was plated (with three replicates per sample) on PAF-S media. The supernatant from the control group (mock-treated) and that from pure stock solution were also plated as negative and positive controls, respectively. All procedures were performed in a laminar flow cabinet. The cultures were incubated at 28^°^C for 2 d, and the number of colonies was counted. The OD of the supernatant was measured, as described earlier.

### Data analysis

Data were analyzed using IBM SPSS (Statistical Package for the Social Sciences), version 17.0 data editor software (SPSS, Chicago, IL, United States). The normality of data was checked by using the Kolmogorov–Smirnov test of composite normality (α = 0.05). Homogeneity of variance was checked by using the Levene test (α = 0.05). If test assumptions were not met, data were taken square root or log-transformed. One-way analysis of variance (ANOVA) and multiple comparisons of differences between treatments were then performed by Tukey’s *post hoc* multiple range (α = 0.05). Different lowercase letters indicate significant differences between treatments (*p* < 0.05).

## Results

### Biological efficacy of *Paenibacillus polymyxa* LMG27872 on *Meloidogyne incognita* parasitism in tomato is dose-dependent

**FIGURE 2:**
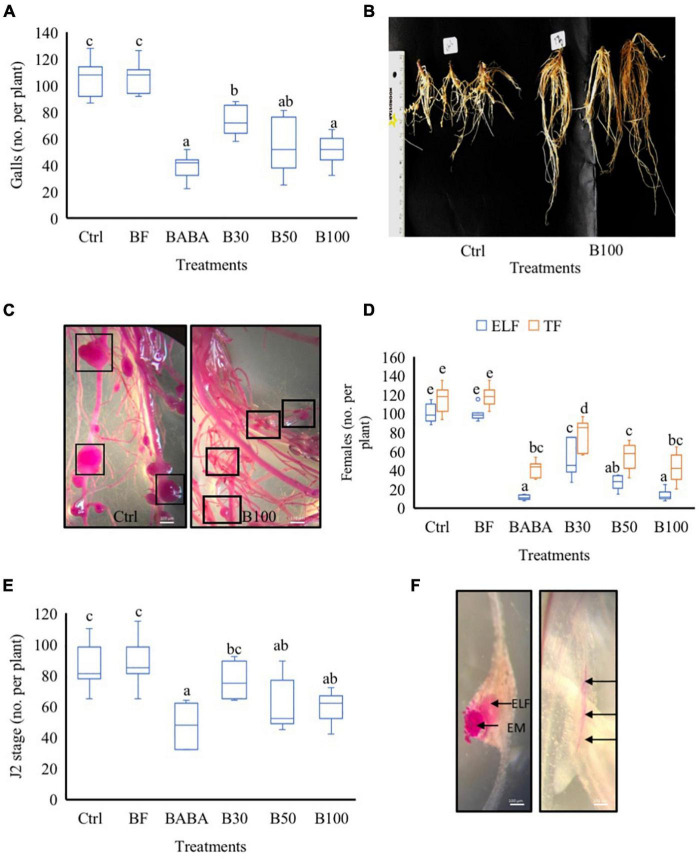
Effect of *P. polymyxa* LMG27872 strain on *M. incognita* parasitism in tomato. **(A)** Total number of galls at 42 dpi. **(B)** Nematode-parasitized roots of the control group and B100-treated plants at 42 dpi. **(C)** Acid fuchsin-stained roots showing gall sizes in the control group vs. B100-treated plants at 42 dpi. **(D)** Number of total females (TF) and the number of egg-laying females (ELF) at 42 dpi. **(E)** Total number J2 at 2 dpi and **(F)** acid fuchsin-stained roots of ELF embedded inside the gall, egg mass (EM) on the surface, and J2 inside the roots (depicted with black arrows). Data were analyzed by one-way ANOVA, followed by Tukey’s *post-hoc* test, and data are shown by box plots with median, upper quartile, and lower quartile. Different letters indicate statistically different means at 95% confidence. Each treatment was replicated eight times (*n* = 8), and each experiment was repeated at least two times. Bars = 100 μm.

To evaluate the biological efficacy of *P. polymyxa* LMG27872 on *M. incognita* parasitism in tomato, 3-week-old plants were treated with BS *via* soil drenching, while control plants were mock- and buffer-treated (Ctrl and BF, respectively). The number of galls on the root system was counted at 42 days post-inoculation (dpi). In comparison with the control group (mock-treated), significantly lower numbers of gall, with 25, 35, and 48% reduction, were observed in plants treated with 30, 50, and 100% BS, respectively (Figure 2A). Plants treated with a high dose (100% BS) exhibited a much stronger effect (25% more effective) than those treated with a low dose (30% BS) (Figure 2A). In addition, smaller galls were observed in plants treated with 100% BS, while mostly large galls were observed in mock-/buffer-treated control groups (Figures 2B,C). To evaluate the effect of *P. polymyxa* on *M. incognita* reproduction, the number of total females (TF) and ELF was counted. In comparison with the control group (mock-treated), a significantly low number of TF was observed, with 29, 47, and 55% reduction in plants treated with 30, 50, and 100% BS, respectively (Figure 2D). A notably much stronger and a dose-dependent effect on ELF was observed with a 55, 66, and 83% reduction (Figure 2D). The effect of 50 and 100% BS was at least two- to three-fold stronger in reducing the number of ELF than the 30% BS. Since *P. polymyxa* LMG27872 had an effect on nematode susceptibility (galls and egg mass at 42 dpi), in the subsequent experiment, the effect of BS dose was evaluated on the J2 stage (effect on penetration) and J3/J4 (developmental stages) at 2 and 7 dpi, respectively. In comparison with the control (mock-treated), the number of J2 was significantly reduced by 21 and 32% in plants treated with 50 and 100% BS (Figure 2E). The ELF is embedded inside the gall and penetrated J2 inside the roots are shown in Figure 2F. At 7 dpi, the number of J3/J4 decreased with increasing doses of BS, but this was not significant (Supplementary Figure 1). Furthermore, at all-time points, significantly lower numbers of nematodes were observed in the positive control (BABA) than in the mock-treated plants (Figures 2A,D,E), validating the parasitism experiments performed using *P. polymyxa* LMG27872. Furthermore, to evaluate if *P. polymyxa* LMG27872 colonized the treated plants, an assay was performed with non-parasitized plants at 1, 3, and 7 days after BS treatment. As shown in Supplementary Figure 2, bacterial colonies were detected in PAF-S plates of tomato roots after 3 and 7 days of BS treatment, with supernatant ODs of 0.3 and 0.5, respectively. No colonization was observed at 1 day after BS treatment.

### *Paenibacillus polymyxa* LMG27872 effect on tomato growth is dose-dependent

**FIGURE 3:**
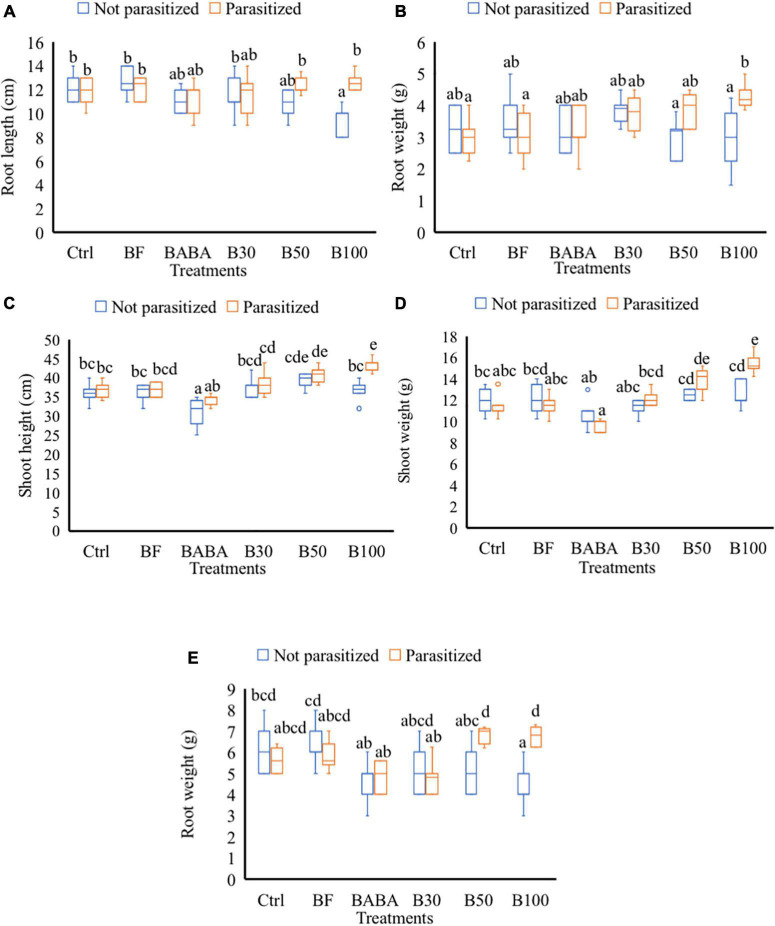
Effect of *P. polymyxa* strain LMG27872 on tomato growth. A subset of plants (8) from each treatment were inoculated with c. 300 second-stage juveniles of *M. incognita* (parasitized groups) and a subset of plants were mock-inoculated (not parasitized groups). Data show the effect on plant growth, **(A)** root length, and **(B)** fresh root weight at 2 days post-inoculation (dpi) corresponding to 9 days posttreatment (dpt), **(C)** SH, **(D)** fresh shoot weight, and **(E)** fresh root weight at 42 dpi/49 dpt, respectively. Data were analyzed by one-way ANOVA, followed by Tukey’s *post-hoc* test, and data are shown by box plots with median, upper quartile, and lower quartile. Different letters indicate statistically different means at 95% confidence.

Treatment with different doses of BS, with or without nematodes, did not cause any significant increase in SH, SW, RL, and RW in comparison with mock-treated plants at 2 dpi/9 dpt (data not shown). At 7 dpi or 14 dpt, a significantly reduced RL (25% reduction) was observed in non-parasitized plants treated with 100% BS when compared to the mock-treated (control not parasitized) tomato plants (Figure 3A). When these (100% BS) treated plants were challenged with nematodes, no negative effect (reduced RL) was observed in comparison with the control parasitized group (Figure 3A). In addition, significantly increased RW was observed in 100% BS-treated + parasitized plants in comparison with ctrl plants (Figure 3B). No effect of 30 and 50% BS was observed on RL and RW for both non-parasitized and parasitized plants. At 7 dpi/14 dpt, no significant changes were observed in SH and SW of plants treated with 30, 50, and 100% BS when compared to control parasitized and non-parasitized groups, respectively (data not shown). At later time points (42 dpi/49 dpt), a dose-dependent effect on growth parameters was observed in BS-treated + parasitized plants. An increase of 10 and 14% on SH (Figure 3C) and 19 and 25% increase in SW (Figure 3D) was observed in 50 and 100% BS-treated + parasitized plants, respectively, when compared with parasitized control plants. No effect of 30% BS was observed on SH and SW for both non-parasitized and parasitized groups (Figures 3C,D). A significant reduced RW (33% reduction) was observed in plants treated with 100% BS when compared to mock-treated (ctrl) non-parasitized plants (Figure 3E). However, when the (100% BS) treated plants were challenged with nematodes, the negative effect (reduced root weight) was not observed in comparison with the parasitized control plants (Figure 3E).

**FIGURE 4:**
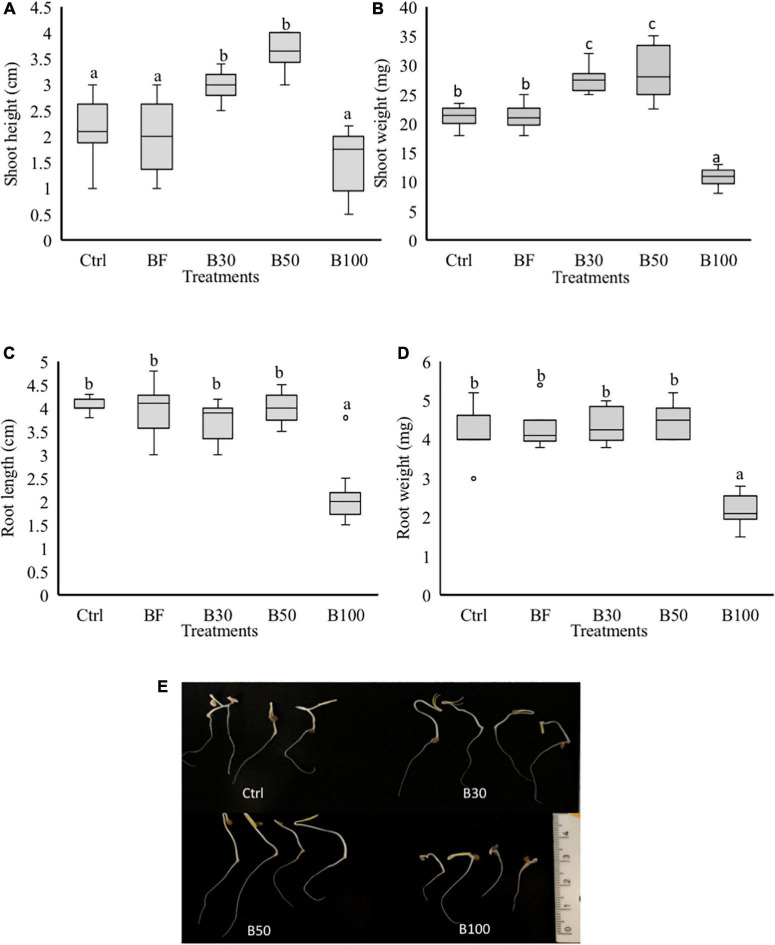
Phenotypic effect of *P. polymyxa* LMG27872 on tomato seedlings. Treatment of 3-day-old seedlings with 1 mL of bacterial inoculum at different bacterial suspension doses of 30, 50, and 100% was performed, while control plants were mock- and buffer-treated. The **(A)** SH, **(B)** SW, **(C)** RL, and **(D)** RW measured at 5 days after application. **(E)** Shoot and root growth of 5-day-old seedlings treated with 30, 50, and 100% BS. Data analyzed by one-way ANOVA, followed by Tukey’s *post-hoc* test, and data are shown by box plots with median, upper, and lower quartiles. Different letters indicate statistically different means at 95% confidence.

To test whether *P. polymyxa* affects plant growth, an *in vitro* experiment was set up to evaluate the effect of *P. polymyxa* LMG27872 on plant growth. A significant increase of 30 and 42% in the SH (Figures 4A,E) and 23 and 25% in SW (Figures 4B,E) was observed in seedlings treated with 30 and 50% BS, respectively, at 5 dpt when compared to the control plants. On the other hand, a significant decrease of 48, 50, and 50% in the SW (Figures 4B,E), RL (Figures 4C,E), and RW (Figures 4D,E) was observed in seedlings treated with 100% BS, respectively, at 5 dpt compared to control plants (Figure 4A). The effect of *P. polymyxa* LMG27872 on tomato growth was dose-dependent. While a high dose of bacteria alone affected plant growth, this negative effect was not observed when these bacterium-treated plants were challenged with nematodes. The inhibitory or stimulating effect of the bacteria on tomato growth *in vitro* also depended on the dose of BS used.

### Effect of *Paenibacillus polymyxa* LMG27872 on hatching and J2 mortality of *Meloidogyne incognita*

**FIGURE 5:**
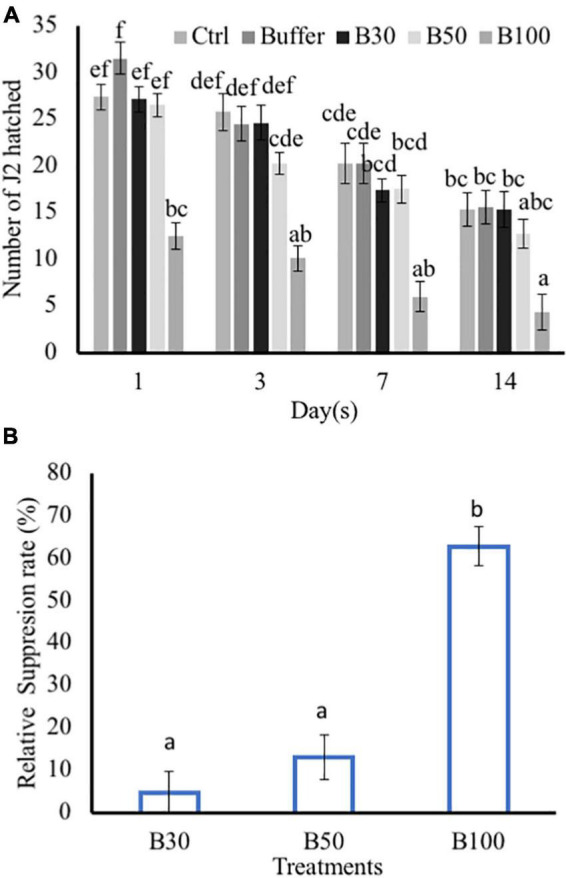
Effect of *P. polymyxa* strain LMG27872 on hatching of *M. incognita in vitro.* The effect of different bacterial suspension doses (30, 50, and 100%) on hatching of *M. incognita* eggs. **(A)** Number of J2 hatched counted at respective time points and **(B)** relative suppression rate of *M. incognita* egg hatching. Data were analyzed by one-way ANOVA, followed by Tukey’s *post-hoc* test; data are shown by box plots with median, and upper and lower quartiles. Different letters indicate statistically different means at (95% confidence).

Exposure to *P. polymyxa* LMG27872 100% BS significantly inhibited *M. incognita* egg hatching (55% reduced J2) in comparison to the control at day 1 (Figure 5A). At day 3, *P. polymyxa* 100% BS significantly inhibited *M. incognita* egg hatching (59% reduced J2) in comparison to control at days 7 days (70% reduced J2) and 14 (72% reduced J2). Although a slight effect of 30 and 50% BS was observed on hatching, it was not significantly different from that of the control (Figure 5A). The relative suppression rate of *M. incognita* egg hatch was calculated over the time course of 14 days, and the relative suppression rate was 5, 13, and 63% in 30, 50, and 100% BS, respectively. The high dose of 100% BS was 12- and 5-fold more effective than 30 and 50% BS, respectively (Figure 5B). The high dose of 100% BS was selected and tested for its effect on *M. incognita* J2 mortality. Within 6 h, there was no effect of 100% BS on J2 mortality, while positive control significantly increased the *M. incognita* J2 mortality, with 18% dead juveniles (Table 1). An effect of 100% BS on J2 mortality was observed at 12- and 24-h exposure time, with mortality of 14 and 60% when compared to the control at respective time points. In general, the effect of BS was visible mostly at 24 h, whereby *M. incognita* J2 were significantly inhibited by *P. polymyxa* LMG27872.

**TABLE 1:** Effect of *P. polymyxa* LMG27872 on J2 mortality (*in vitro* test).

Treatment	Number of J2 dead and mortality % at different exposure time											
	6 h				12 h				24 h			
	Mean no. of dead J2	±SE	Siglevel	Mortality (%)	Mean no. of dead J2	± SE	Siglevel	Mortality (%)	Mean no. of dead J2	± SE	Siglevel	Mortality (%)
Water	2	0.3	a		2	0.6	a		2	0.6	a	
Buffer	2	0.3	a		2	0.7	a		3	0.6	a	
B100	3	0.7	a	3.0	14	1.1	b	14	60	3.2	e	60
Velum prime	18	3.0	b	18.0	36	3.0	c	36	43	1.5	d	43

A total of 100 freshly hatched J2 added into cavity blocks with only high concentration (100%), control (water and buffer), and positive control fluopyram (Velum^®^ Prime) 1.0 μg/mL in the dark at 23^°^C. The motility of J2 was monitored under a dissecting microscope at 6, 12, and 24 h post-incubation. Dead J2 count data analyzed by one-way ANOVA followed by Tukey’s post-hoc test. Different letters indicate statistically different mean 95% confidence for the number of dead J2. Percent mortality calculated as (dead J2/total J2) × 100) at respective time point of exposure.

## Discussion

RKNs are the most damaging PPN group. The pot experiments showed that *P. polymyxa* LMG27872 inhibited *M. incognita* parasitism in tomato in a dose-dependent manner. Reduced numbers of galls, egg masses, and J2s were observed in all BS-treated plants, with 100% BS-treated plants exhibiting a much stronger effect on nematode penetration, development, and reproduction. Similarly, application of a BS of *P. polymyxa* GBR-1 (Khan et al., 2008) and KM2501-1 (Cheng et al., 2017) into potting soil reduced the *M. incognita* root galls and the number of nematodes in a dose-dependent manner in tomato. The biological efficacy of *P. polymyxa* was also observed in other crops. A greenhouse experiment on carrots with *P. alvei* T30 resulted in significantly reduced galls and the egg mass index of *M. incognita* (Viljoen et al., 2019). The reduced susceptibility observed with *P. polymyxa* LMG27872 was comparable with positive control RS BABA. RS BABA-induced resistance has previously been described to be effective in tomato against *M. incognita* (Devran and Baysal, 2018) and *M. javanica* (Oka et al., 1999). *Paenibacillus polymyxa* displayed antimicrobial activity against many other plant pathogens, such as hairy root disease, caused by rhizogenic *Agrobacterium* in tomato (Vanlommel et al., 2020; Vargas et al., 2021a,b, and antifungal activity in peanuts (*Arachis villosa*) (Costa et al., 2021) and hemp (*Cannabis sativa*) (Mahmoud and Jabaji, 2021). Other *P. polymyxa* varieties such as NMA1017 have shown to exert biocontrol activity against *Rhizoctonia solani* on maize (*Zea mays*) *in vitro*, while pot experiments with bean (*Phaseolus vulgaris*) exhibited the growth of fungal pathogens such as *R. solani* and *Pythium ultimum* (Chávez-Ramírez et al., 2020).

A beneficial effect was also reported against *Phytophthora tropicalis*, on cacao (*Theobroma cacao*) (Chávez-Ramírez et al., 2021). In contrast to the findings obtained in our research, *P. polymyxa* LMG27872 showed limited biocontrol activity against the fungal pathogen *Botrytis cinerea* on strawberry (*Fragaria ananassa*) and against *Rhizoctonia solani* on maize (Langendries, 2022). The type of assays, dose, host plants, and pathogens can affect the biological efficacy of this endophytic bacterium. Nevertheless, several *in vitro* and *in vivo* studies have demonstrated the potential of several bacteria and fungi as microbial control agents for PPNs (Kumar and Dara, 2021).

Endophytic bacteria are known to produce antimicrobial metabolites, toxic VOCs which can have direct effects on nematodes. *P. polymyxa* is known to produce an array of compounds and traits with effects on plant growth, biofertilization, biocontrol, and protection against abiotic stresses (Langendries and Goormachtig, 2021). Results from the *in vitro* assays showed direct effects of *P. polymyxa* on J2 mortality, comparable with the commercial nematicides used as a control. This is in line with previous research whereby *in vitro* experiments showed *M. incognita* (Khan et al., 2008; Viljoen et al., 2019) and *M. graminicola* (Bui et al., 2020) J2 mortality when exposed to *Paenibacillus* species.

Endophytic bacteria can directly attack, kill, immobilize, or repel PPNs as they find their host, compete for space, and produce secondary metabolites and other toxic VOCs, such as flavonoids, peptides, quinones, alkaloids, steroids, phenols, terpenoids, and polyketones, or lytic enzymes such as chitinases, cellulases, hemicellulases, and 1,3-glucanases (Gamalero and Glick, 2020; Kumar and Dara, 2021). The differences in the observed phenotypes with different doses of BSs could be due to dilution of already low concentration of the compound/substance present in the suspension. Only further investigations of present compounds in different dilutions can elucidate this. Some endophytic bacteria are also known to produce hydrogen cyanide, suppressing RKNs such as *M. incognita* by inhibiting mitochondrial respiration (Abd El-Rahman et al., 2019). On the other hand, endophytic bacteria can suppress PPNs indirectly through competition for nutrients, inducing systemic resistance (ISR) and triggering accumulation of hydrogen peroxide and ascorbic acid (Hasky-Guenther et al., 1998; Oka et al., 1999; Reitz et al., 2000; Siddiqui and Shaukat, 2002a; Choudhary et al., 2007; Lee et al., 2012; Vos et al., 2012; Li and Chen, 2019; Gamalero and Glick, 2020). Further investigation on the mode of action of *P. polymyxa* suppressing *M. incognita* can shed more light on our findings. Genomic analyses of *P. polymyxa* LMG27872 showed genes associated with metabolites, such as rhizomide genes encoding chitinases (Langendries, 2022). These metabolites are known to have antimicrobial properties and could explain its biological efficacy. *Paenibacillus polymyxa* produces avermectin, polymyxin, fusaricidin, and polyketide, which protect plants against pathogens and are involved in activation of ISR (Abd El Daim et al., 2015; Rybakova et al., 2017; Park et al., 2018; Li and Chen, 2019; Chávez-Ramírez et al., 2020). Pot experiments performed here also showed reduced numbers of J2 in *P. polymyxa*-treated plants in a dose-dependent manner. Less J2 in the roots could be explained by the direct effect of *P. polymyxa* on the soil by means of VOCs. Also, the formation of biofilms on roots could hamper penetration. *Paenibacillus polymyxa* is known to form tight biofilms around the root, which produce exopolysaccharides, protecting from pathogenic bacteria and fungi (Haggag and Timmusk, 2008; Timmusk et al., 2009, 2019; Abd El Daim et al., 2015; Luo et al., 2018; Yi et al., 2019). It is also known to repel *M. incognita* J2 (Hu et al., 2017) when *P. polymyxa* attaches to the cuticle of *M. hapla* and activates PAMP-triggered immunity (PTI)-responsive genes in tomato roots, reducing the J2 establishment, confirming an

ISR (Topalović et al., 2019, 2020). *In vitro* tests showed that *P. polymyxa* had an inhibitory effect on hatching of *M. incognita* in a dose-dependent manner. Similarly, exposure of *M. incognita* to *P. polymyxa* GBR-1 under *in vitro* conditions significantly reduced egg hatching in a dose-dependent manner (Khan et al., 2008). Endobacteria are known to inhibit PPNs through direct antagonism with synthesis of lytic enzymes (Gamalero and Glick, 2020). Chitinase produced by *P. illinoisensis* KJA-424 caused the lysis of *M. incognita* eggshells and resulted in the inhibition of hatching *in vitro* (Jung et al., 2002).

In addition to the observed dose effect of *P. polymyxa* on tomato susceptibility to *M. incognita*, a dose-dependent effect of the bacterium on tomato growth was also observed. Increased growth linked to the use of the endophytic bacterium has been observed in tomato (Katsenios et al., 2021), barley, cucumber, pepper, sesame, and *Arabidopsis thaliana* (Jeong et al., 2019). Plant growth-promoting rhizobacteria (PGPR) are known for production and degradation of the phytohormone auxin, leading to enhanced root growth (Leveau and Lindow, 2005; Spaepen et al., 2014; Finkel et al., 2020). The beneficial effect on growth relies on diverse mechanisms, such as acquiring limiting nutrients, that is, phosphate solubilization (Turan et al., 2012), oxidation of sulfur into sulfate (Grayston and Germida, 1991), production of siderophores to capture iron (Marschner et al., 2011), and nitrogen fixation (Hurek and Reinhold-Hurek, 2003; Iniguez et al., 2004). *Paenibacillus polymyxa* increases soil porosity, fixes nitrogen, and solubilizes phosphorus (Lal and Tabacchioni, 2009; Lal et al., 2016). The isolate MVY-024 was found to significantly increase the amount of ammonium and mineral N in the soil, thus promoting plant growth. However, phytotoxic effects have also been reported (Maes and Baeyen, 2003; Timmusk et al., 2005; Hong et al., 2016). *In vitro* studies showed that *P. polymyxa* LMG27872 inhibits maize seed germination in a dose-dependent manner (Maes and Baeyen, 2003).

We also showed a negative effect of *P. polymyxa* LMG27872 on root and shoot phenotype of tomato. However, in the pot assay, the negative effect on growth was absent when the plants were challenged with *M. incognita*. The mechanism observed here coincides with a faster and stronger defense response following pathogen attack, a phenomenon termed as defense priming (Conrath et al., 2015; Martinez-Medina et al., 2016; De Kesel et al., 2021). A primed defense response was observed in other studies using endophytic bacteria and fungi to control *M. incognita* (Vos et al., 2013; Hu et al., 2017; Dahlin et al., 2019; Abd El-Aal et al., 2021). Negative effects of BSs on growth could be linked to genes related to plant cell wall degradation. Endoglucanases, glycoside hydrolases, and xylanases were found in the genome of the bacterium used in this research (Langendries, 2022). Future experiments can be performed to test whether diffusible and/or VOCs affect plant growth.

The tests performed using *P. polymyxa* showed both direct (nematicidal effects) and indirect effects. This suggest that its mode of action for the biological control of *M. incognita* may be paralysis and antibiosis, which caused mortality, immobility, prevention of invasion, and inhibition of hatch. Based on this, we suggest double application: one application at seeding or planting, and a second application when egg masses are being formed. Given the fact that soil application will further dilute the BS, 100% BS is recommended.

## Conclusion

In conclusion, results showed, for the first time, a biological efficacy of *P. polymyxa* LMG27872 on *M. incognita* attacking tomato, and this effect was dose-dependent. The effect of *P. polymyxa* on tomato growth was also dose-dependent. The highest dose of BS had a negative effect on plant growth. However, such negative effect was not observed when the BS-treated plants were challenged with *M. incognita*, suggesting tolerance or a defense priming mechanism. The promising results show the potential of *P. polymyxa* LMG27872 to protect plants from RKN parasitism and its implementation in integrated nematode management suitable for organic production.

## Data availability statement

The raw data supporting the conclusions of this article will be made available by the authors, without undue reservation.

## Author contributions

RS and WW planned and designed the research. RS conducted parasitism experiments and *in vitro* tests and wrote the manuscript. WW read, commented, and approved the manuscript. Both authors contributed to the article and approved the submitted version.
