# Supplementary figures and images for: Endophytic Paenibacillus polymyxa LMG27872 inhibits Meloidogyne incognita parasitism, promoting tomato growth through a dose-dependent effect
Source: Front Plant Sci. 2022 Sep 14;13:961085. doi: 10.3389/fpls.2022.961085 (PMC9516289; doi:10.3389/fpls.2022.961085)

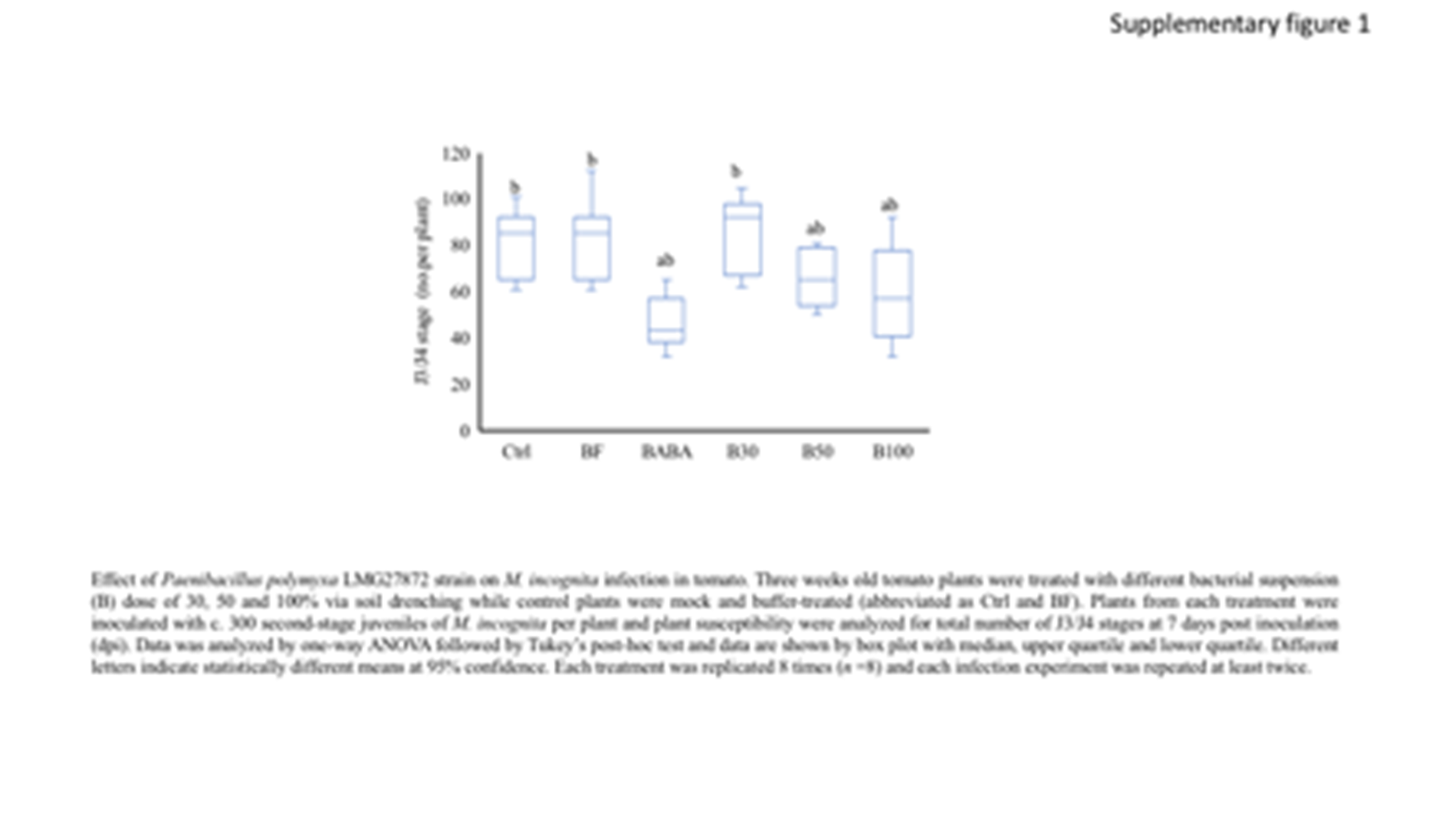

Supplement: Supplementary file 1 [file Image_1.tiff]

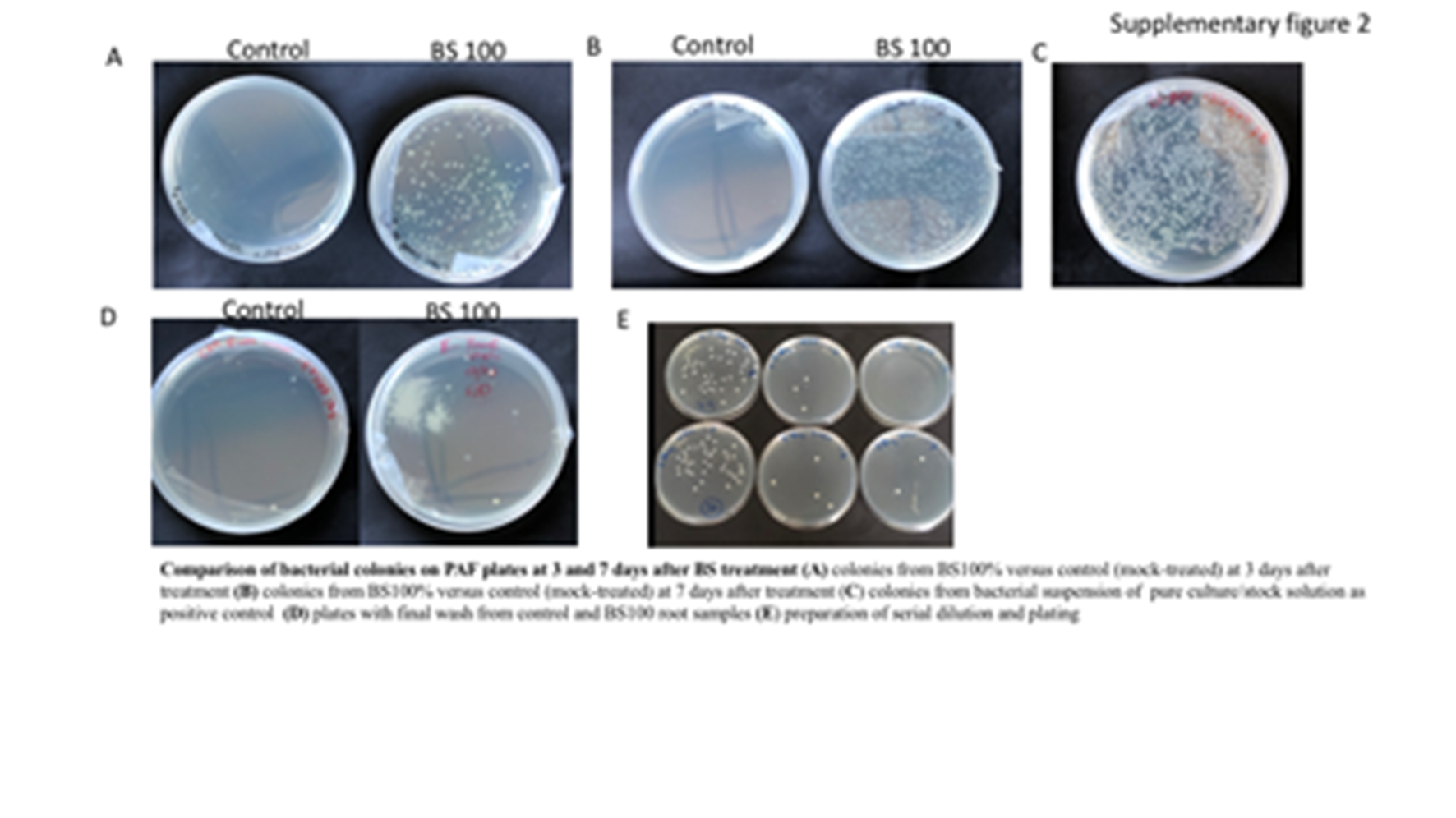

Supplement: Supplementary file 2 [file Image_2.tiff]
